# Editorial: VR, AR, MR in healthcare: the role of immersive technologies in medical training

**DOI:** 10.3389/fdgth.2025.1669899

**Published:** 2025-08-14

**Authors:** Serena Ricci, Veronica Penza, Francesco Neri

**Affiliations:** ^1^ Department of Informatics, Bioengineering, Robotics and System Engineering, University of Genoa, Genoa, Italy; ^2^ Simulation and Advanced Education Center - SimAv, University of Genoa, Genoa, Italy; ^3^ Biomedical Robotics Lab, Advanced Robotics, Istituto Italiano di Tecnologia, Genoa, Italy; ^4^ Department of Medicine, Surgery and Neuroscience, University of Siena, Siena, Italy

**Keywords:** virtual reality, mixed reality, extended reality, medical training, medical education, healthcare simulation, augmented reality

**Editorial on the Research Topic**
VR, AR, MR in healthcare: the role of immersive technologies in medical training

Immersive Technologies are increasingly used in several fields of medical education. Currently, there is a multitude of Virtual Reality (VR) and Augmented Reality (AR) applications, both commercial and research prototypes, covering the entire study path of nursing and medical students (1). Such applications range from tools to study physiology and anatomy (2), to simulators to train and test technical skills, procedural knowledge and soft skills (3, 4). VR and AR have been proved to be effective in many specialties including surgery, emergency medicine, radiology, obstetrics and dentistry and many more (5, 6).

Several reasons have supported the use of immersive technologies in medical education; adult learning theories promote their use for the training and evaluation of medical students, as they provide a repeatable, and learner-friendly environment (7). Moreover, these technologies provide riskless, controlled and personalized tools to train theoretical, manual and non-technical skills simultaneously. Additionally, Extended Reality (XR) applications make simulation-based education affordable and accessible anywhere, as they do not require dedicated simulation centers (8, Neri et al.).

The goal of this Research Topic is to offer a perspective on the use of immersive technologies in medical education, through multidisciplinary contributions about new applications, reviews and efficacy studies. Two new systems have been proposed: a novel affordable user interface for robotic surgery training (Neri et al.), and a proof of concept of an AR application for the training of hemorrhage management (Tharun et al.).

The use of VR in surgery has becoming increasingly popular, especially for the training of laparoscopic skills and recently also for robotic-assisted surgery. Within this framework, VR simulators could offer a solution to acquire technical skills; however, the high cost potentially limits their spread in surgical residency schools. The user interface proposed by (Neri et al.) aims to overcome the cost barrier by fully simulating the console both in terms of functions and ergonomics. Additionally, the system enables haptic feedback through haptic devices. According to the review on VR and AR in medical education submitted to this research topic (Tene et al.), “*the integration of haptic feedback technology marks an important advancement in medical training*” and therefore it should be further explored. However, validation studies carried out on expert surgeons and naïve subjects revealed that haptic feedback provided during simple preparatory tasks did not affect performance, even though participants found it useful. Conversely, in a pure virtual environment experts outperform naïve participants, suggesting that the simulator resembles a real Da Vinci console (Neri et al.).

Another simulator presented in this Research Topic is an AR proof of concept of a system for the training of massive hemorrhage management (Tharun et al.). Conversely to the robotic-assisted surgery simulator (Neri et al.), this tool is targeted to non-experienced users and explores the possibility of providing personalized feedback through a multimodal approach. The system simulates an out-of-hospital emergency requiring the rescuer to position an antihemorrhagic device onto a manikins' limb. According to the user's actions, the system provides different types of cues such as images, text and oral instructions. Due to the heterogeneity of the feedback, a usability study has been carried out revealing a good usability, user experience and low workload; these results suggest that interactive and tailored cues are appreciated by trainees and should be further explored to heads toward an increasingly personalized training (Tharun et al.). During the experiment, some subjects reported difficulties in interacting with the application, particularly pressing buttons and handling virtual objects. This is a common challenge which can potentially limit the usage and the training potentialities of immersive technologies (9).

To ensure that trainees can effectively interact with the virtual environment, it is advisable to include a familiarization phase before the simulation. Indeed, this approach might reduce the cognitive load and guarantee that neither users' performance nor their ability to learn is impacted by unfamiliarity with the system (10). The work of (Vittadello et al.) investigated the role of familiarization. By asking participants whether they require a familiarization with the virtual environment before simulation, it emerged that such phase should be included into VR-based simulation This result is in agreement with (Tharun et al.) that includes a familiarization section in the AR application. Specifically, users could decide how long to run the demo before starting the simulation. Regardless of participants' previous experience with VR/AR, all users familiarized with the system for a similar amount of time, suggesting that a familiarization section is effective and appreciated by trainees.

Other than surgery and emergency medicine that show many examples of Mixed Reality training applications, XR can be also used for diagnostic and interventional education purposes. The review presented by (Tene et al.) investigated the role of immersive technologies for medical and radiation physics education. This discipline involves both the application of physics principles to medical imaging and the use of radiations for diagnosis and therapy; therefore, its applications are broad and include software to train procedural knowledge in radiology, surgical simulators for imaging-guided procedures, and patients responding to radiations therapies (Tene et al.).

Even though a variety of applications for medical training exist, less than 25% of them have been evaluated for performance-based outcomes and only four out of ten have undergone a usability test (Tene et al.). Despite this lack of validation, many applications are now integrated into educational settings, and several proof of concepts and prototypes will be soon part of medical education curricula (Tene et al.). Indeed, current evidence suggests that XR application for medical training boost skills retention and foster the empathy of healthcare providers as well professionals' communication skills. Additionally, XR can promote patient-centered care, by providing clinicians visual instruments to study complex cases and inform patients about treatment options and outcomes (11). Within this exciting framework, there is a need for ongoing multidisciplinary research involving computer scientists, engineers, clinicians, pedagogists, and psychologists to keep pace with technological advancements and validate existing solutions. Simultaneously, facilitators should increase the use of immersive technologies in medical education. This would in turn improve the overall quality of the available simulators, enhance patient safety, and increase the research in the field, ultimately closing the loop between medical education and technological development.

## References

[1] Mergen M Graf N Meyerheim M. Reviewing the current state of virtual reality integration in medical education-a scoping review. BMC Med Educ. (2024) 24(1):788. 10.1186/s12909-024-05777-5 39044186 PMC11267750

[2] Hammouda SB Maoua M Bouchahma M. The effectiveness of VR-based human anatomy simulation training for undergraduate medical students. BMC Med Educ. (2025) 25(1):816. 10.1186/s12909-025-07402-5 40452030 PMC12128225

[3] Savir S Khan AA Yunus RA Rehman TA Saeed S Sohail M Virtual reality: the future of invasive procedure training? J Cardiothorac Vasc Anesth. (2023) 37(10):2090–7. 10.1053/j.jvca.2023.06.032 37422335

[4] Dubiel A Kamińska D Zwoliński G Ramić-Brkić B Agostini D Zancanaro M. Virtual reality for the training of soft skills for professional education: trends and opportunities. Interact Learn Environ. (2025) 33(5):3261–81. 10.1080/10494820.2025.2450634

[5] Ricci S Calandrino A Borgonovo G Chirico M Casadio M. Virtual and augmented reality in basic and advanced life support training. JMIR Serious Games. (2022) 10(1):e28595. 10.2196/28595 35319477 PMC8987970

[6] Wu Q Wang Y Lu L Chen Y Long H Wang J. Virtual simulation in undergraduate medical education: a scoping review of recent practice. Front Med (Lausanne). (2022) 9:855403. 10.3389/fmed.2022.855403 35433717 PMC9006810

[7] Reed S Shell R Kassis K Tartaglia K Wallihan R Smith K Applying adult learning practices in medical education. Curr Probl Pediatr Adolesc Health Care. (2014) 44(6):170–81. 10.1016/j.cppeds.2014.01.008 24981666

[8] Li X Elnagar D Song G Ghannam R. Advancing medical education using virtual and augmented reality in low-and middle-income countries: a systematic and critical review. Virtual Worlds. (2024) 3:384–403. 10.3390/virtualworlds3030021

[9] Ingrassia PL Mormando G Giudici E Strada F Carfagna F Lamberti F Augmented reality learning environment for basic life support and defibrillation training: usability study. J Med Internet Res. (2020) 22(5):e14910. 10.2196/14910 32396128 PMC7251481

[10] Young JQ Van Merrienboer J Durning S Ten Cate O. Cognitive load theory: implications for medical education: AMEE guide no. 86. Med Teach. (2014) 36(5):371–84. 10.3109/0142159X.2014.889290 24593808

[11] Jones D Fealy S Evans D Galvez R. The use of extended realities providing better patient outcomes in healthcare. Front Med (Lausanne). (2024) 11:1380046. 10.3389/fmed.2024.1380046 38439895 PMC10909988

